# Traditional use of *Garcinia kola* Heckel in Nigeria – the results of questionnaire survey compared with scientific evidence

**DOI:** 10.1186/s12906-026-05287-5

**Published:** 2026-02-16

**Authors:** Uyai-Abasi Otuekong Ukut, Eszter Csikós, Nóra Papp, Dragica Purger

**Affiliations:** Department of Pharmacognosy, Faculty of Pharmacy, University of Pécs, Pécs, Hungary

**Keywords:** Bitter kola, Ethnobotany, Ethnomedicine, Kolaviron, West Africa

## Abstract

**Background:**

Bitter kola, *Garcinia kola* Heckel, is a woody plant native to West and Central Africa. It is traditionally used both in social ceremonies and for treating a wide range of ailments, including malaria, respiratory infections, gastrointestinal disturbances, hepatitis, metabolic and sexual disorders. Despite its importance and popularity, a great deal of information and basic knowledge is still missing about the species. Particularly lack of studies is related to therapeutic efficacy and safety of bitter kola uses.

**Aims:**

This study aimed to document ethnomedicinal uses of bitter kola among native populations in Nigeria, to identify commonly used plant parts and the dosages, record perceived side effects and to compare these findings with scientific evidence, and to estimate its potential clinical applications.

**Methods:**

Data were collected by a structured questionnaire distributed both online and in-person to members of the Nigerian public.

**Results:**

A total of 152 respondents participated, primarily from the Ibibio, Annang, and Igbo ethnic groups. Most participants reported using fresh seeds of bitter kola, mainly for treating respiratory symptoms, stomach upset, and general well-being. Regular use was common, typically, monthly or as needed, depending on the disease.

**Conclusions:**

Several traditional uses of bitter kola are supported by clinical studies: including its antioxidant and immunomodulatory effects and its benefits for gastric, hepatic, glycemic, respiratory, reproductive and pain-modulation related conditions. Evidence also suggests antimicrobial and hepatoprotective potential. However, further studies are required to establish standardised dosages and safety profiles for different plant parts.

**Supplementary Information:**

The online version contains supplementary material available at 10.1186/s12906-026-05287-5.

## Background

Traditional cultures around the world have dedicated time to recognizing the healing properties of plants in their environments, as they are custodians of knowledge, closely related to their lands, which hold their identity. Ethnobotanical studies documenting these indigenous practices related to plants have played a significant role in the discoveries and development of treatments for both communicable and non-communicable diseases [1–4]. In West Africa one of the most culturally and medicinally significant species is *Garcinia kola* Heckel, commonly known as bitter kola, belonging to the Clusiaceae (formerly Guttiferae) family [5]. This species is distributed in the forest zones of West and Central Africa, including Ghana, Sierra Leone, and Nigeria [6]. It is locally called Efit by the Annang people of South Nigeria. Historically, the earliest record mention bitter kola in West Africa appears in an 1889 book, a manual of economic botany describing its local use for rheumatism, and its export for soap production in Europe [7].

**Fig. 1:**
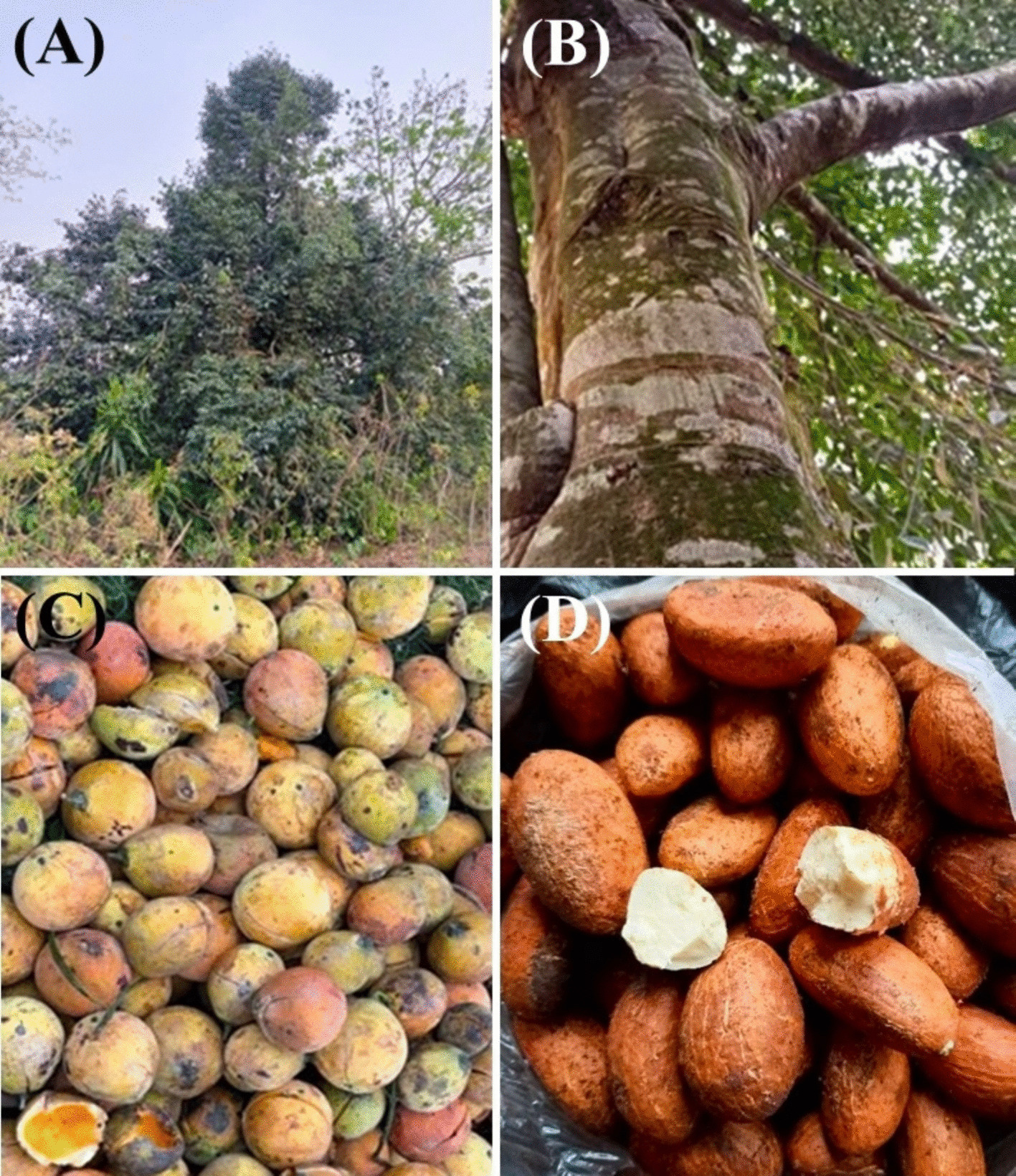
Garcinia kola. **A** Crown with leaves; **B** Trunk and bark; **C** Fleshy fruit; **D** Seeds (Photo by Sonny Ukut)

Bitter kola is an evergreen tree with a dense, broad crown that can grow up to approximately 20 m in height. Its trunk is straight, with brown bark that produces a resinous gum, enhancing water resistance (Fig. 1A, B). The leaves are elliptic, leathery, with entire edges. The flowers have four greenish-white petals [8]. The fleshy fruit contains two seeds, or "nuts" (Fig. 1C, D).

*Garcinia kola* is listed as one of six preferred tree species by the World Agroforestry Centre (ICRAF) for domestication in West and Central Africa [9]. The species is currently classified as “vulnerable” in IUCN’s Red List of Threatened Species mainly due to habitat loss as a consequence of continuous deforestation practices, overexploitation of the tree from the wild and slow-growing seedlings [10].

Phytochemicals with potential clinical relevance are present in the entire plant, including the seed, fruit, stem, root, and bark. These parts contain chromanols, such as garcinal, garcinoic acid, and δ-tocotrienol, which contribute to plant’s antioxidant activities [11, 12]. Flavonoids and bioflavonoids—including kolaflavanone, Garcinia bioflavonoid-1 (GB-1), GB-2, GB-1a, and kolaviron (mixture of GB-1, GB-2, and kolaflavanone) are also present in the plant [13, 14]. Additional compounds such as saponins, alkaloids, cardiac glycosides, tannins, and phenols contribute to the plant’s antimicrobial properties [15, 16]. Garcionic acid and kolaviron have been investigated for anti-inflammatory effects, particularly in atherosclerosis and knee osteoarthritis [17–19]. Furthermore, catechin, garcinol, garcionic acid, and d-tocotrienol have important role in the treatment of erectile dysfunction [20]. The ability of bitter kola to exhibit medicinal effects is attributed to its bioactive compounds, such as kolaviron and garcinol, which exhibit strong antioxidant and immunomodulatory activities. This compound has been shown to neutralize reactive oxygen species, modulate cytokine production and influence transcription factors involved in inflammatory and metabolic pathways [21–27].

This plant holds deep cultural and social significance across West and Central Africa. It is used as a flavoring agent, stimulant, and beverage ingredient, and it plays an important role in social gatherings; as a greeting symbol, to welcome guests in a traditional hospitality ceremony, and in various healing rituals [28]. Among the Igbo ethnic group of eastern Nigeria, the seeds are offered to guests as a simbol of peace and goodwill [29]. Certain communities such as the Nsukka people of south-eastern Nigeria use the seeds as protection against perceived spiritual poisonings [30], and to expel snakes from their hiding place [31]. The plant is also used traditionally to treat different health conditions, including cough, hypertension, hepatitis, tumour, and insulin-dependent diabetes, as well as ketotic states. Midwives have used it in pregnancy, often combined with *Cocos nucifera* L. milk and palm oil from *Elaeis guineensis* Jacq. for treatment of oedema and toxaemia [32]. Bitter kola has also been used for bacterial and viral infections [33], as well as for bronchitis, gonorrhoea, and food poisoning [34]. In Nigeria, several ethnic groups prepare a decoction of the root, seed, stem, and bark to treat respiratory conditions such as cough and asthma [35–37]. In Cameroon, the bark is used for treatment of diarrhoea [38]. Some groups also consume the seeds, bark or root with local alcoholic beverages for aphrodisiac or intoxicating effects [39]. The latex of the plant is applied to fresh wounds to prevent sepsis [6] and used for parasitic skin diseases [32]. Traditional herbalists employed the seed for tuberculosis treatment [40]. The roots serve as chewing sticks for oral hygiene [41]. A polyherbal formulation including *Garcinia kola* with *Moringa oleifera* Lam. and *Aloe vera* (L.) Burm. f. *barbadensis* is used as a traditional treatment of acquired immunodeficiency syndrome (AIDS) [32].

Scientific studies corroborate many of these traditional medicinal uses. Extracts of bitter kola suppress proinflammatory genes iNOS and COX-2. [18] and produce antinociceptive and anti-inflammatory effects through opioidergic and adrenergic pathways [42]. In reproductive health seed consumption has been associated with increase hormone levels and improved male fertility. However, studies also report disruption of oestrous cycle in the experimental female rats [22]. Cardiovascular studies indicate that bitter kola seed extract reduces arterial blood pressure and increases basal heart rate with further cardioprotective effects, including inhibition of caspase 3 (pro-apoptotic enzymes), improvement of cell survival signalling in ischemia-injured rats, mitigation of pathological cell proliferation, vasodilatation, increase of antioxidant defences, and reversal of cardiac damage caused by doxorubicin [18, 26, 43–47]. In respiratory conditions seed extracts demonstrate antitussive effect [48], bronchodilatation through inhibition of 5-lipoxygenase-mediated phospholipid metabolism [49], and antibacterial activity [50–52]. In asthma models seed extracts reduces the tracheal wall thickness and mucus plugging [49]. The bitter kola exhibits hepatoprotective activity by restoring of liver enzyme profiles [53, 54]. In gastrointestinal conditions, bitter kola prevents inflammatory cell infiltration and thickening of the gastric wall [55]. Neuroprotective effects have also been documented: extracts prevent cognitive and motor decline following the acute radiation exposure, inhibit neuroinflammation of the hippocampal region by suppress oxidative stress, reduce the neuronal damage [56–63]. Finally, the plant demonstrates chemoprotective potential by reducing lipid peroxidation, restoring of antioxidant levels, mitigation oxidative DNA, proteins, and lipid damage, inhibiting stress-response proteins, and suppress cancer-related transcription factors [64–66].

Several systematic reviews on bitter kola appeared recently (e.g. [34, 67, 68]. Despite its importance and popularity, a great deal of information and basic knowledge about the species is still missing. Particularly few studies are related to therapeutic efficacy and safety of bitter kola uses. There is a lack of attempts aimed at evaluation of overlapping of traditional knowledge and contemporary medicinal practice.

The present study aimed to document the ethnomedicinal practices related to bitter kola among native communities in Nigeria, to identify commonly used plant parts and the dosages, record perceived side effects and compare these findings with existing scientific evidence to estimate the therapeutic applicability of bitter kola.

## Methods

### Questionnaire survey

**Fig. 2:**
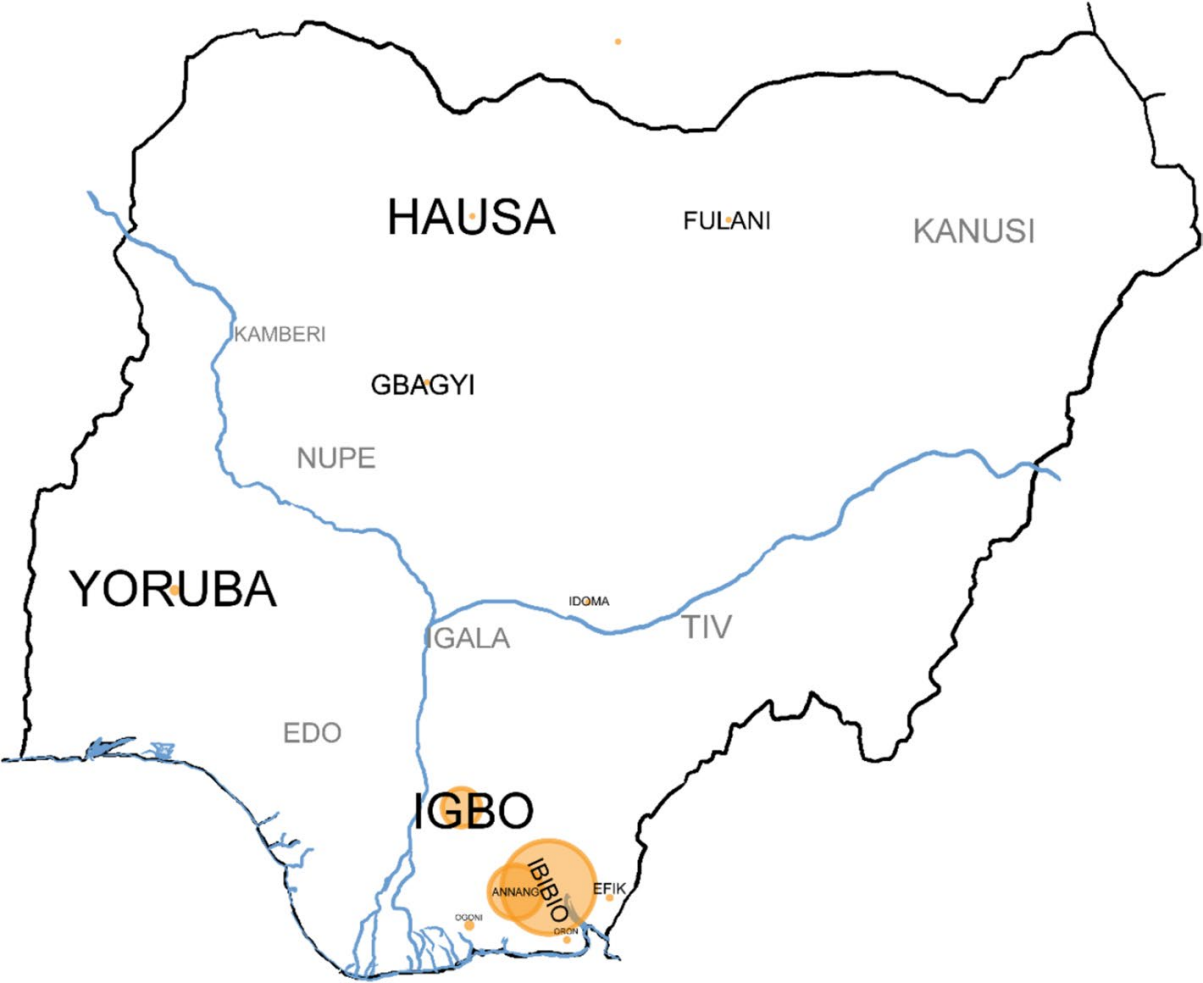
Ethnic groups in Nigeria (based on [71]) – The population size—larger font size, circle size—number of responses per tribe

To document the traditional use of bitter kola, a questionnaire was developed following guidelines and the recommendations from the ethnobotanical literature [69, 70]. The survey in the summer of 2023 was distributed online and in person to indigenous people of South-South Nigeria (Fig. 2. All respondents received the same questions, which included demographical information (age, sex, ethnicity, place of origin, and residence; questionnaire provided in supplementary material. Most questions addressed the traditional uses of bitter kola. Since this plant is a native species in the rainforests of West Africa, it was assumed that most of indigenous respondents would be familiar with and make use of the plant.

During manual distribution and in-person interviews priority was given to the community elders and middle-aged individuals. Participants who contributed to this study included members of the Women’s Fellowship Committee of the Methodist Church Nigeria, Ayanya Circuit; scientists at the Ministry of Science and Technology, Akwa Ibom state; The Paramount ruler and elders of Ikot Udoe, in Ikot Ekpene Local government area (LGA). After survey distribution, respondents were given time to complete the questionnaire, and it was collected in batches to ensure anonymity.

Prior informed consent was obtained from all participants, and community permission was granted by the relevant traditional authorities. The study adhered to the International Society of Ethnobiology (ISE) Code of Ethics.

### Statistical analysis

Qualitative responses were coded thematically and analysed descriptively following standard ethnobotanical data analysis procedures. The data from the questionnaire were copied into an excel table and the basic statistical analysis was carried out using R [72]. A contingency Table (7 indications × 5 plant parts: seed, leaf, bark, root, sap) was analysed to test whether plant-part choice differed by therapeutic indication. Pearson’s chi-square test was used, and a Monte Carlo simulated p-value (20,000 replicates) was reported due to low expected counts in some cells. Effect size was summarized using Cramér’s V.

## Results

A total of 170 questionnaires were distributed to the public, which 125 were returned. Forty responses were excluded from the analysis: 12 were incompletely filled, 16 contained conflicting answers, and 5 respondents reported no knowledge of the bitter kola plant. Additionally, 22 responses were obtained through in-person interviews and 5 via online submission. In total, 152 valid responses were included in the final analysis.

### Demographic profile of the respondents

The 152 respondents represented 12 ethnic groups: Annang (39 persons), Efik (2), Fulani (1), Gbagyi (1), Hausa (1), Ibibio (68), Idoma (1), Igbo (28), Ogoni (4), Oron (2), and Yoruba (4) from the south-south, south-east, south-west, the north-east and -central regions of Nigeria, and 1 respondent from southern Niger Republic of the Muzu tribe (Fig. 2).

With respect to settlement types, 55% of all respondents live in the cities, 29% in towns, 12% in villages, and 5% of respondents live in suburban areas. The proportion of male and female individuals in the sample was almost equal (77 and 75, respectively). Most respondents were young adults, generally well educated (Table 1).

**Table 1:** Demographic profile of the respondents

Variable	Group	Individual response	Proportion of respondents (%)
**Gender**	Female	77	51%
	Male	75	49%
**Education**	Primary school	1	1%
	Secondary school	44	9%
	Technical collage	14	29%
	Bachelor	87	57%
	PhD	6	4%
**Settlement types**	Village	17	11%
	Town	44	29%
	Suburb	7	4.6
	City	84	55%

### Traditional use of bitter kola

**Fig. 3:**
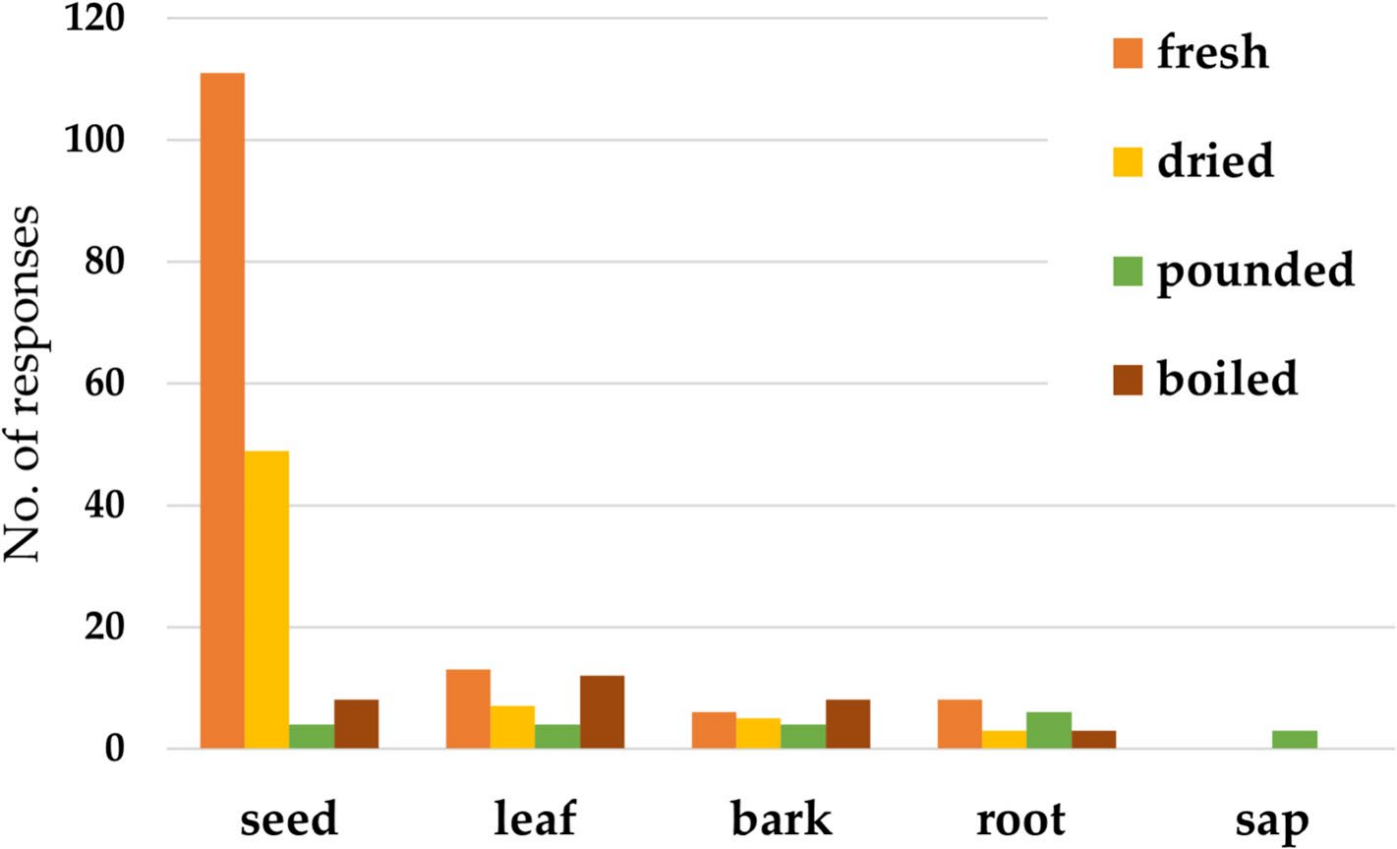
Forms of bitter kola’s parts used

Respondents were first asked which part of bitter kola they consumed orally on a non-medicinal, leisurely basis. Seeds were the most commonly consumed part of *G. kola* reported by 97% of respondents. In contrast, leaves, bark, and roots were consumed to a far lesser degree (9%, 7% and 5% respectively), while 3% of individuals reported not consuming any plant part. No responds reported leisurely ingestion of sap, suggesting that it is not used orally in non-therapeutic contexts. Consumption pattern showed that respondents typically ingest 1–3 seeds either fresh or dried. Leaves and bark were usually prepared fresh or boiled, commonly taken as a drink or inhaled as steam. Roots were chewed fresh or pounded for preparation, while sap was typically extracted fresh and strictly pounded (Fig. 3). A few respondents described more elaborate preparations, such as grinding seeds into water or palm wine (native gin) until the fluid turns white.

The duration of personal use of bitter kola in relation to their age (Table 2) highlighted that younger respondents (13–18 years) had used the plant only for 1–3 years, whereas respondents aged 19–25, and 26–30, reported about up to 10 years of experience with bitter kola. In the questionnaire there was an option provided for respondents who cannot remember when their first experienced using bitter kola: whole life: In the sense that they have been using bitter kola for as long as they can remember, regardless of their age. Respondents who replied that they never use plant parts could be because bitter kola is not taken leisurely, still when prescribed by a relevant authority figure, or they are simply among the 3% of respondents who did not use bitter kola.

**Table 2:** The age of the respondents and the period of the use of bitter kola

Period of use of bitter kola	13–18 years	19–25 years	26–30 years	31–50 years	51–70 years	Above 70 years
Never	0	4	0	1	0	0
As needed	0	1	0	0	0	0
Few weeks	0	2	2	1	0	0
Few months	2	11	2	5	0	0
1–3 years	3	14	6	8	0	0
5 years	0	0	0	1	0	0
10 years	0	15	11	16	1	0
20 years	0	7	7	12	1	0
More than 30 years	0	0	0	9	4	1
More than 50 years	0	0	0	0	1	0
Whole life	0	0	0	1	2	1

Regarding the frequency of use of different plant part, seeds, leaves, bark are used on a monthly basis, while roots are personally used yearly. The consensus relays that the plant parts are used as needed or recommended by a traditional healer.

Analysis of the usage of bitter kola plant parts in relation to ethnic groups revealed that seeds were the most used plant part amongst all represented ethnic groups. Leaves, bark, and root are utilized to a lesser degree (by the Annang, Efik, Ibibio, Idoma, Igbo, and Yoruba respondents) or not utilized (Fulani, Gbagyi, Hausa, Oron, and Muzu respondents) (Table 3).

**Table 3:** Used parts of bitter kola reported by respondents from different ethnic groups

Ethnic group	Seed	Leaf	Bark	Root	Sap	None
Annang	39	9	9	7	2	0
Efik	2	1	1	1	0	0
Fulani	1	0	0	0	0	0
Gbagyi	1	0	0	0	0	0
Hausa	1	0	0	0	0	0
Ibibio	67	9	6	4	0	1
Idoma	1	0	1	0	0	0
Igbo	24	7	8	5	1	1
Ogoni	4	1	0	1	0	0
Oron	2	0	0	0	0	0
Yoruba	4	2	2	2	2	0
Muzu (from Niger)	1	0	0	0	0	0

#### Cultural importance of bitter kola

Overall, 48% of respondents stated that utilize bitter kola as a symbol of hospitality, whereas 52% did not use the plant seed for welcoming guests. Ethnic differences were evident (Fig. 2): Efik, Gbagyi, Hausa, and Muzu respondents reported at 100% of usage of bitter kola in guest welcoming costumes. Annang (59%) and Igbo (57%) reported such usage. Meanwhile, Fulani, Idoma, and Oron respondents did not use the plant at all. Among Ogoni and Ibibio people use it 25% and 38% respectively. The responses of Yoruba people revealed a 50% split difference on seed use for guest welcome.

### Ethnomedicinal use of bitter kola

Both males and females used the bitter kola seeds at a similarly high frequency (97% and 96% respectively). Males reported two times more frequent use of roots than females (17% and 9%, respectively), and the sap (5% and 1% respectively). However, statistical analyse did not show significant difference (chi-square = 5.497, df = 4, *p* = 0.2397) between sexes in utilization of plant parts.

#### Preparation of plant parts used

**Fig. 4:**
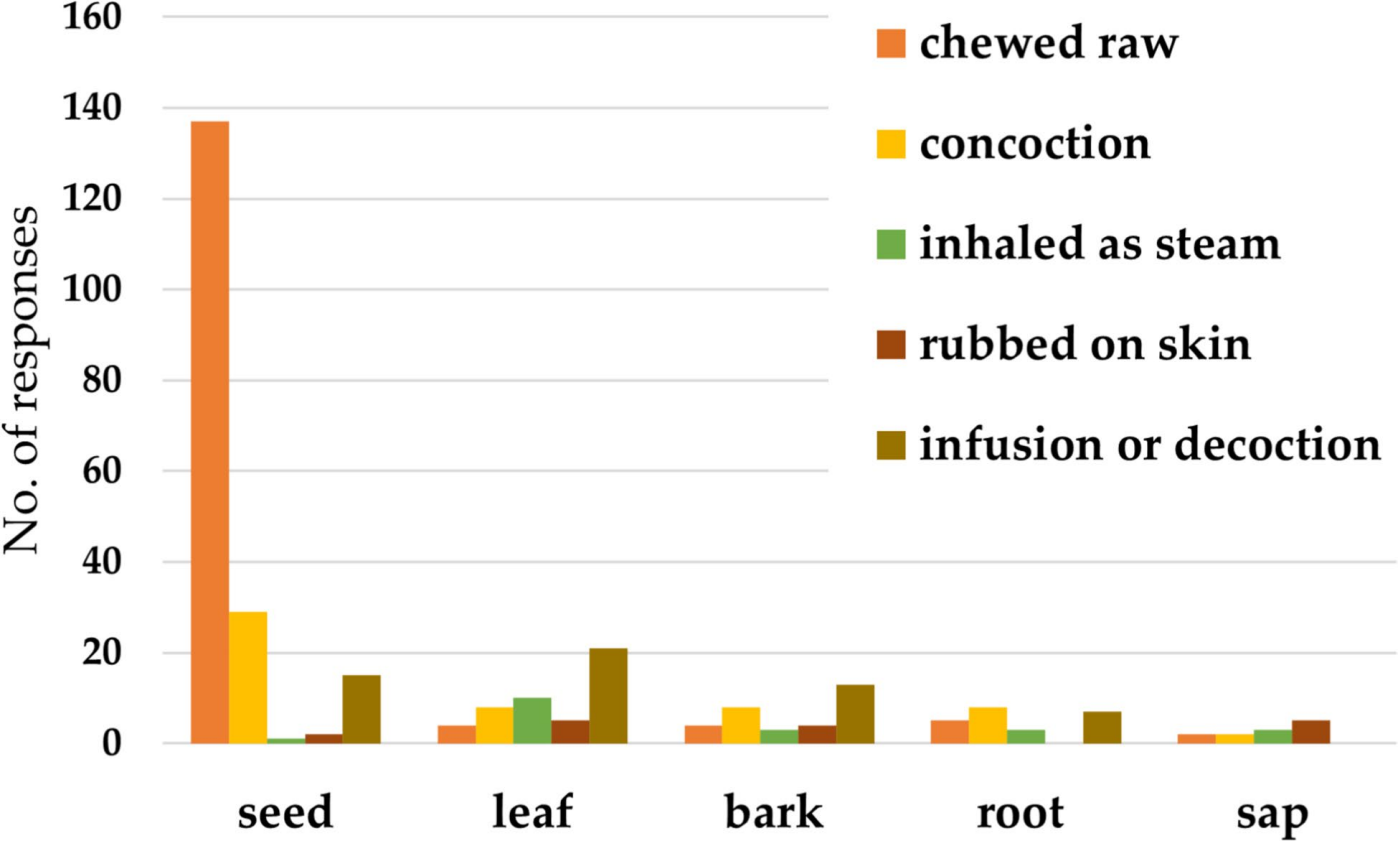
Preparation of parts of bitter kola used in the ethnomedicine in the study area

Seeds were most frequently used either in fresh or dried form for various illnesses (Fig. 3), or prepared as a mixture with local gin, water, or chewed raw. Leaves were used fresh or boiled as tea or as steam inhalation. Bark and roots were used fresh or boiled, so that it can be placed into a concoction or boiled as tea (Fig. 4). Sap was reported to be collected fresh or dried and rubbed on the skin. Respondents described additional practices: chopped seeds, leaves, bark, or roots are placed into water or local gin and left for 1–2 days to saturate the liquid. Some commented that they placed the seed product in a mixture with garlic and honey. Others would chew bitter kola seeds simultaneously with alligator pepper seeds (*Aframomum danielli* (Hook.f.) K.Schum.), or ingest "Tom-Tom", a popular menthol-containing candy, alongside the seeds. In the case of gastric issues, the seed or root is pounded, boiled, filtered, cooled, and poured into the rectum of a child, as an enema.

#### Therapeutic applications

**Fig. 5:**
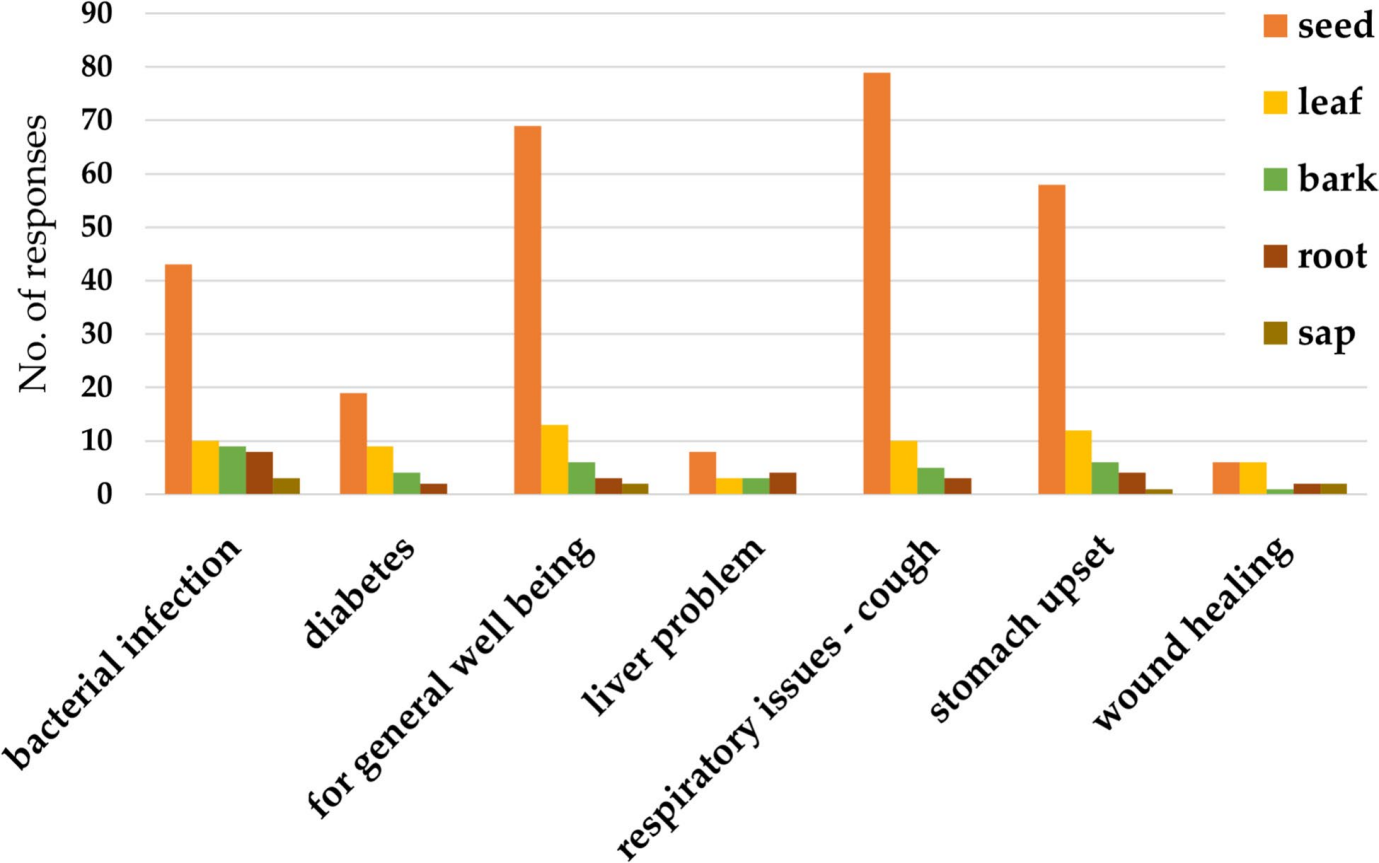
Conditions treated with different parts of bitter kola

Seeds of *Garcinia kola* were dominant plant part used therapeutically, mostly to treat respiratory conditions, in particular, cough (Fig. 5). Leaves are used to supplement one’s general well-being and in the treatment of stomach upset, respiratory conditions, bacterial infection; however, to a lesser degree than seeds. Bark, root, and sap are utilized in the treatment of bacterial infection by fewer respondents, if even used at all (Fig. 5). Further applications were reported by respondents: roots can be used to treat pain and malaria. The bark was used against malaria; dried bark soaked in palm wine is used to treat stomach ulcers. Leaves saturated in water also treat stomach ulcers, and this water is boiled and then used for bathing to treat eczema or inhaled in case of cough or catarrh. A response listed treatment of erectile dysfunction, and antidote for poisons treated with the plant.

Some respondents offered supplementary information as requested and reported some traditional beliefs around the bitter kola plant. For instance, spreading broken or chewed bitter kola seeds in one’s home would deter snakes from the home. The respondents also mentioned a commonly held belief of how the plant neutralizes "charms" (evil intention) and evil spirits. Others included its neutralizing property with venom, its ability to reduce blood sugar levels, and increase sexual stamina. Bitter kola is also stated to aid pregnant women with morning sickness and spitting by reducing these sensations. More insight was given into the utilization of sap: the fresh sap can be soaked in water, after a day, a drop of that water is to be applied as eyedrops as treatment for glaucoma. This protocol can be carried out with the seed to treat eye irritation. Treatment of wounds can be done by using a fluid saturated with seed to cleanse the area. Its neutralizing ability is rumoured to also affect modern medicine, because ingesting bitter kola in combination will 'cancel out' the effect of the prescribed drug.

#### Dosage and treatment duration

**Fig. 6:**
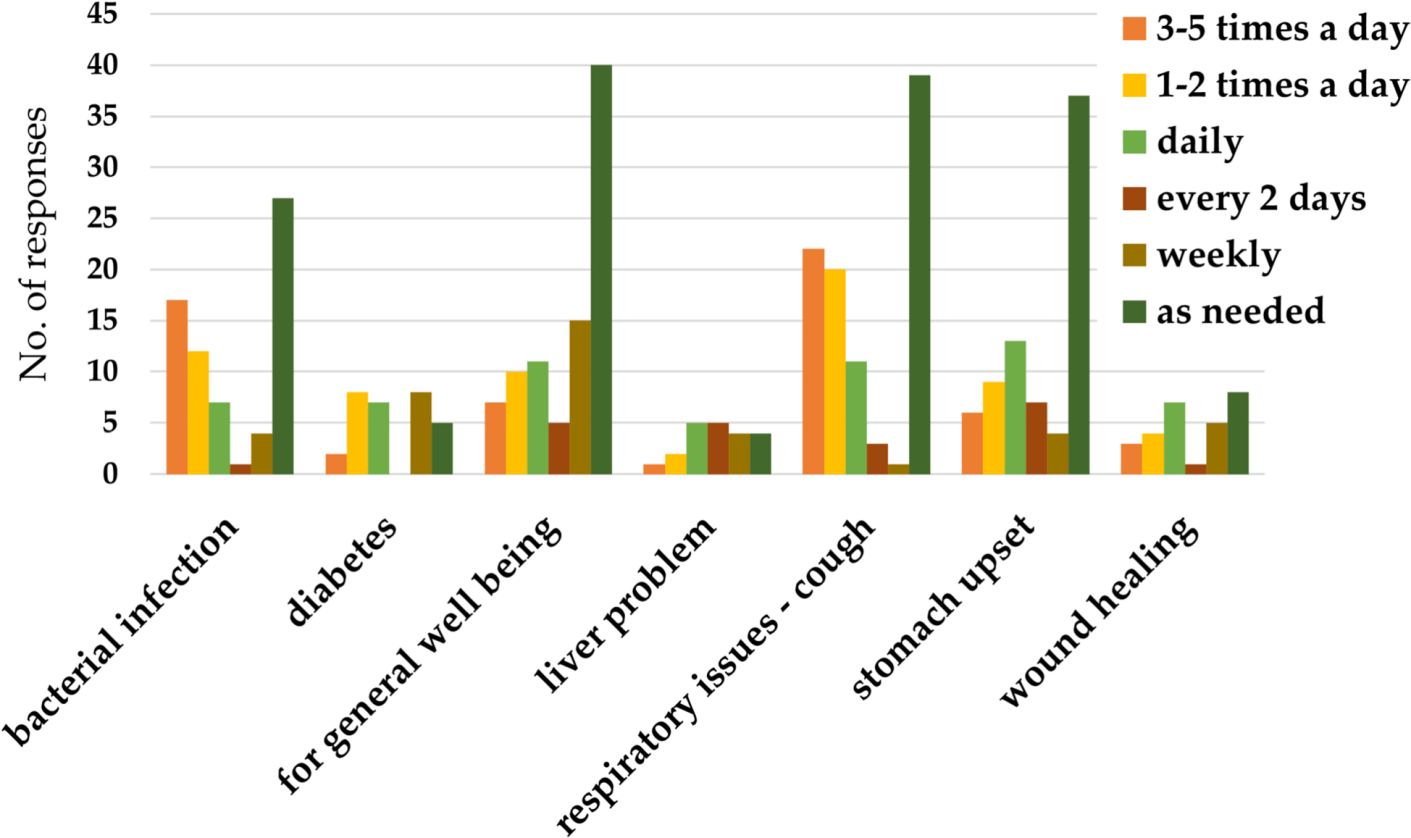
Frequency of use of bitter kola for medical treatment

**Fig. 7:**
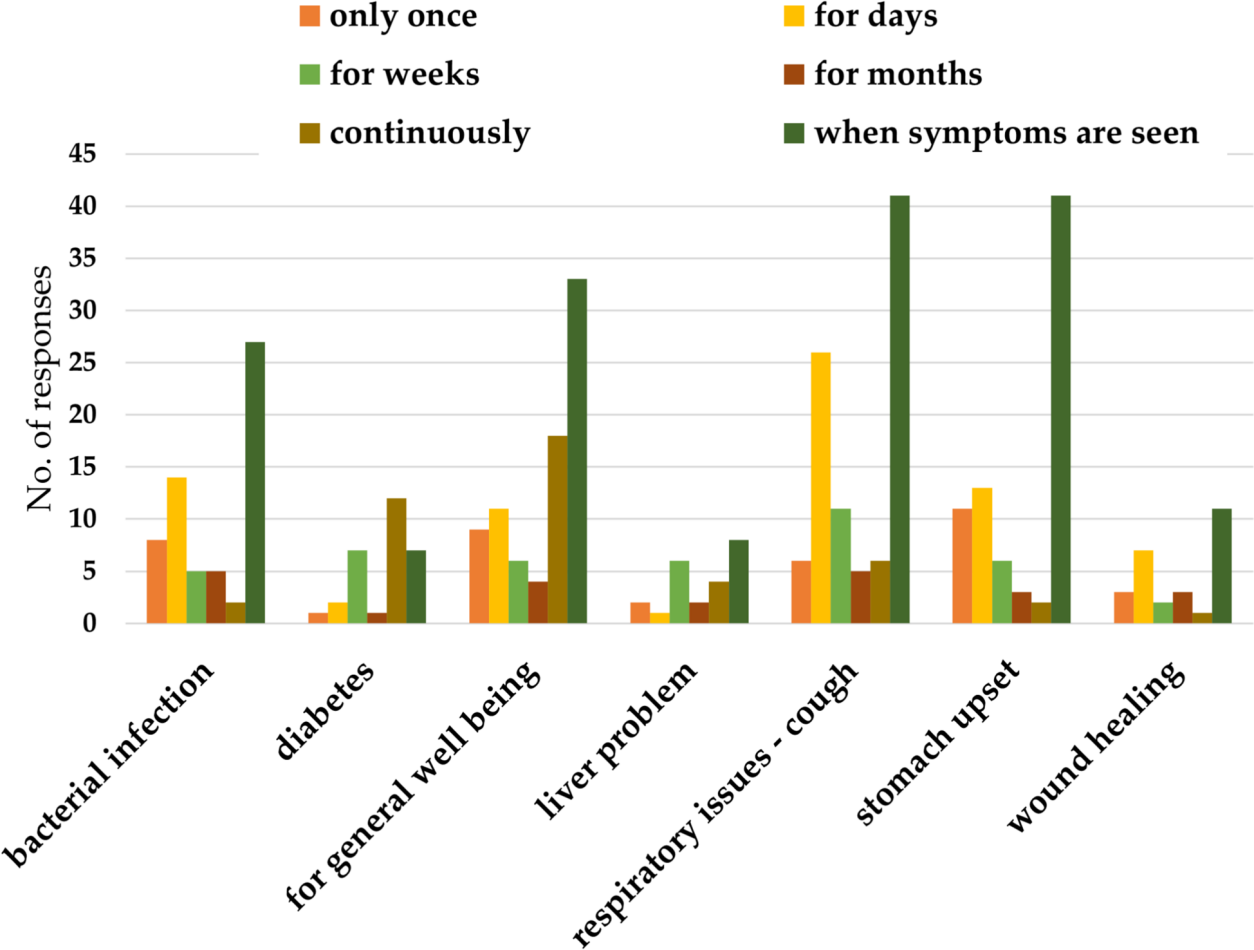
Period of medical use of bitter kola

Typical dosages ranged from 1 to 3 seeds, or approximately 30 ml of liquid medicine which is a "shot". The maximum amount of medicine taken in a day ranged from 1 to 7 seeds or from one to 4 shots per day. Feedback reported that bacterial infections and cough were treated 3–5 times a day, while stomach upset and wound healing treatment were utilized daily (Fig. 6). The duration of treatments depended on the disorder, and most respondents administered remedies when symptoms were seen, thus as needed (Fig. 7).

#### Adverse effects

Most respondents reported no adverse effects. Those who had experienced side effects mentioned that eating a lot of seeds caused one to experience hunger, dry mouth, and teeth discoloration. Other reported side effects include dizziness, diarrhoea, and heartburn.

#### Transmission of knowledge

The survey revealed that parents were the primary source of medicinal plant knowledge (45%), followed by grandparents (29%). Neighbors, traditional healers, and herbal books accounted for 6% of reported knowledge transmission. Regarding usage patterns, self-medication with bitter kola was most common (65%), while 12% of respondents reported treatment of their children or parents, (9%) treated community members.

Additionally, there was little variation between the usage of bitter kola amongst online and in-person respondents. Among online respondents, 60% were within the 19–25-year of age.

### Comparison of traditional with modern medicine

A traditional use of bitter kola reported by respondents in this study was compared with results of in vivo and in vitro studies of the therapeutic applicability of bitter kola (Table 4).

**Table 4:** Comparing the traditional use of *Garcinia kola* with modern scientific evidence

Condition	Traditional use	Modern evidence
Antimicrobial	Bark and root for malaria treatment Seeds are consumed mostly 3–5 times a day, for the period of time symptoms persist, or “for days”	Significantly inhibited parasite load, *Plasmodium falciparum* and *P. berghei* proliferation [73, 74], [75]; [76]. Exhibits antibacterial action against a range of oral, respiratory, gastrointestinal bacterium, viruses, and fungi [52, 77–79]
Diabetes	Seeds and leaves are consumed, 1–2 times a day, daily or weekly continuously	Inhibited enzymes α-amylase and α-glucosidase which decrease carbohydrate breakdown. Significantly decreased serum glucose levels. Increased levels of antioxidant enzymes Glutathione peroxidase (GSH-Px), Superoxide dismutase (SOD), Catalase (CAT), and decreased levels of malondialdehyde lead to inhibition of lipid peroxidation, thus possibly ameliorating diabetic complications [21, 80–83]
Hepatic injury	Seeds and roots are consumed daily or every 2 days for as long as symptoms persist	Restored liver function enzymes to normal levels, improved lipid levels and accumulation, and preserved hepatocyte structural integrity [54, 83]
Respiratory conditions, e.g., cough	Mainly, seeds are used taken 3–5 times a day or as many times as needed. Leaves boiled in water and steam are inhaled to treat a cough	For acute cough, there was a decrease in coughing bouts [48]. Also supported broncho-dilation in asthma [49]
Gastrointestinal issues	Seeds and leaves are used, taken daily, for days or as symptoms persist Dried bark and leaves steeped in palm wine (local gin) for the treatment of stomach ulcer	Preserved epithelial histology of gut exposed to cell-destroying toxicity, prevented inflammation and maintained acid production [55, 84, 85]
Wound healing	Seeds are steeped in water. Solution is used to clean wounds. Seeds or leaves are used as needed or daily for as long as symptoms persist	Inhibited increased expression of iNos, Cox-2, and TNF-α [86], [18]. Also alleviated pain, joint stiffness, and mobility difficulties in patients with osteoarthritis of the knee [17]

## Discussions

This study documented the traditional knowledge of *Garcinia kola* highlighting both cultural and ethnomedicinal practices among group of 152 respondents belonging to diverse ethnic groups in South Nigeria. Respondent in our study were mostly young adults, generally well educated, living in cities and towns, as a consequence of online data collection, which could not include herbalist healers, medicinal practitioners and elders from rural communities. This could have affected the depth of knowledge on bitter kola due to distance from traditional practices. Additionally, it is possible that relatively large proportion of incomplete questionnaires may be consequence of lack of knowledge on traditional uses of bitter kola. However, our study showed that most participants were within an urban context, though still possessed a traditional knowledge and experiences on uses of bitter kola that has been passed down, mainly from their parents and grandparents. Despite the establishment of modern medical facilities in Africa, many people still rely on indigenous herbal remedies, medicines and prefer consultation with traditional health practitioners for their health care [87]. In Nigeria, about 75–80% of both rural and urban populations use traditional medicine [88]. Many ethnobotanical studies have been conducted amongst rural communities; however limited information is available on the indigenous cultural knowledge amongst mixed tribal urban communities [89]. Prinsloo et al. [90] show that indigenous traditional knowledge is still prevalent in urban setting, although the extent of this knowledge was significantly influenced by cultural group, participant age, and age of migration. Collective knowledge within a particular culture or indigenous society is socially constructed [91]. Ethnobotanical knowledge transfer is the generational passing of traditional understanding about plants (identification, uses) within cultures, primarily through oral traditions, observation and practice, from experienced community members (elders, herbalists) to younger generations. The knowledge of how to identify, harvest, prepare, and use plants is a testament to the deep understanding of nature and health possessed by traditional healers and community elders [92–97, 99,98 ]. The cultural and spiritual significance of particular plants fosters a sense of identity and community cohesion. By empowering communities through education and awareness, ethnobotanical knowledge becomes a key element in building resilience against external challenges, ensuring the holistic well-being of local populations. To continue enjoying the benefits of traditional medicine, governments, and regulatory bodies must design and implement guidelines that would protect traditional knowledge, ensure safe practices, and encourage respectful collaboration between traditional healers, researchers, and regulatory authorities [25].

Amongst the genders of respondents some trends were observed of plant part usage (the roots and sap were used by males more), statistical analyses indicated that these differences were not significant.

Although nearly half of respondents used bitter kola as a symbol of hospitality, this practice varied substantially across of ethnic groups. The low representations of some ethnic groups in this sample limited broader generalizations. Among those who retain this practice were respondents from Annang, Efik, Gbagyi, Hausa, and Muzu ethnicity. There was a split among Yoruba respondents on the utilization of bitter kola seed, suggesting that the variability was tied to family or community level rather than a uniform ethnic tradition. This study corroborated using of bitter kola seeds as a hospitality symbol by the Igbo people [29], but showed that the other southern Nigerian tribe, Ibibio and Oron did not utilize the seed in this way. This was surprising, as it is colloquially known as a southern hospitality habit.

Bitter kola seeds are used medicinally against infections chewed raw or prepared as aqueous or alcoholic solution and taken as needed or 3–5 times a day, for as long as symptoms are seen. This aligns with scientific findings illustrated broad spectra of potential antibacterial, antifungal, and antiparasitic effects [41, 73, 76, 79, 100]. Ranging from oral pathogens [79], gastrointestinal and respiratory pathogens [50, 51], it also has an immune-modulating response to combat viruses [52]. Respondent reported frequent dosing (3–5 times daily or as needed), reflects the rapid onset treatment paradigm commonly described in traditional medicine for acute infections.

The traditional use of bitter kola for diabetes was consistently reported in the study. Fresh or dried bitter kola seeds were used, chewed raw, continuously for weeks. Extensive biochemical studies corroborate this practice: the anti-diabetic effect of the seeds could be due to kolaviron, that reduces carbohydrate breakdown by inhibiting enzymes α-glucosidase and α-amylase, lowers fasting glucose levels, and prevents oxidative stress-induced diabetic complications [21, 80, 82, 101, 102]. Iwu et al. [13] suggested the extract’s insulin-like activity, even in models, further supports its potential utility in treatment of diabetes.

Liver-related ailments were also treated with seeds and, to lesser extent, roots. The respondents typically used fresh seeds daily, or every 2 days, when symptoms were evident.

Experimental studies strongly support the hepatoprotective activity of bitter kola seeds through normalization of liver enzymes, mitigation of oxidative stress and maintaining hepatic cellular structure [46, 53, 54, 103, 104]. These finding align with participant reports of improved symptoms like dark urine and general detoxification.

Respiratory conditions, particularly cough were treated mainly with fresh seeds, with leaves employed as steam inhalations. Scientific evidence confirms that bitter kola seed reduces the frequency of cough [48] and facilitates bronchodilation and smooth muscle relaxation via inhibition of Ca^2+^ mobilization, and histamine release [49]. The plant’s demonstrated antiviral and anti-inflammatory properties [50–52], further strengthen its relevance for respiratory conditions.

Respondents in this study reported varying treatment approaches for gastric ailments, including ulcers, food poisoning, and diarrhoea. Mainly fresh or dried seeds, leaf extracts and bitter kola bark soaked in palm wine were used. Pharmacological studies have shown gastroprotective effects of these bitter kola’s ailments through antioxidant stabilization of epithelial structures, and inhibition common pathogenic bacteria, such as *Salmonella* and *Vibrio* species [55, 77, 105, 106]. The congruence between reported practices and laboratory findings is therefore substantial.

The wound healing was the least commonly cited use of bitter kola amongst participants of our survey. Scientific evidence supports anti-inflammatory effects of bitter kola seed extract, and its ability to suppress expression of proinflammatory genes [42], [18].

Respondents provided also knowledge regarded crushed bitter kola plant’s perceived ability to repel snakes, neutralize poisons and “charms” and reduce the potency of modern medicine. Bitter kola snake-repelling effect, although reported in other traditional cultures [31], currently lack empirical testing. While previous observations and studies reported bitter kola’ interactions with some pharmaceutical agents, e.g. Quinine sulfate, an antimalarial drug [107], the mechanisms of these claims remain unverified. These beliefs represent important directions for future research in chemical ecology and pharmacokinetic interactions. There is a strong correlation between the traditional medicinal uses of *Garcinia kola* and modern scientific evidence, with many traditional claims supported by in vitro and animal studies, although a lack of human clinical trials means its therapeutic efficacy in humans is not specified [39]. Human clinical trials should be performed with the extracts or compounds from *G. kola* in the future with the potential for the drug development. More toxicological studies and clinical trials of bitter kola and its compounds are required to avoid any adverse effects of their possible clinical use [68, 108, 109].

Limitations of our study were defined by the demographic characteristics of our sample. We had no representative number or respondents for several ethnic communities and for general demographic structures of villages, therefore our findings may not be necessarily generalizable to other populations in Nigeria. Nevertheless, the strong overlap between traditional practices and experimental evidence underscore potential of *Garcinia kola* as multisystem therapeutic agent. Future studies should prioritize clinical trials assessing efficacy and safety in humans; standardization of dosage, especially for seeds and alcoholic extracts; toxicological profiling across age groups and with chronic use; investigation of plant–drug interactions, particularly with antimalarial and antidiabetic medications; evaluation of its role against multidrug-resistant pathogens.

## Conclusion

Seeds of bitter kola were consistently identified as the most widely used plant part, typically consumed to treat respiratory conditions, gastrointestinal disturbances, infections and to boost general wellbeing. Traditional practices generally involved frequent administration over the duration of symptoms, rather than fixed dosing schedules. Comparison with in vitro and in vivo studies revelled strong pharmacological support for traditional uses of bitter kola in treatment of bacterial infection, diabetes, wounds, hepatic injuries, gastric disorders, and respiratory ailments. However, more research should be performed to establish the dosage and toxicity level of the plant parts used.

## Supplementary Information

Supplementary Material 1

## Data Availability

The datasets used and analysed during the current study are available from the first author on reasonable request.

## Abbreviations

ALP: Alkaline phosphatase
AST: Aspartate Aminotransferase
ALT: Alanine aminotransferase
DNA: Deoxynucleic Acid
COX-2: Cyclooxygenase-2
GB-1/1-a/2: Garcinia bioflavanoid-1/1-a/2
TNF-α: Tumor Necrosis Factor- alpha
