# Supplementary material for: Traditional use of Garcinia kola Heckel in Nigeria – the results of questionnaire survey compared with scientific evidence
Source: BMC Complement Med Ther. 2026 Feb 16;26:109. doi: 10.1186/s12906-026-05287-5 (PMC13014730; doi:10.1186/s12906-026-05287-5)
Supplement: Supplementary file 1 — Supplementary Material 1 [file 12906_2026_5287_MOESM1_ESM.pdf]

Evaluation of the traditional use of *Garcinia kola* Heckel in West Africa, the modern scientific evidence, and clinical use potential

INTRODUCTION

Garcinia kola Heckel, bitter kola is a woody plant from Clusiaceae family, native to West and Central Africa. It is utilized traditionally in greeting ceremonies and in treatment of a variety of ailments such as malaria, bacterial infections, gastrointestinal disturbances, hepatitis, glycemic and reproductive disorders. Its wide range of use warrants scientific investigation to validate or disprove its effects.

This is an anonymous survey to collect information on the traditional medicinal uses of bitter kola. The collected data will be used in my dissertation (Thesis) to show the ethnobotanical use of the bitter kola plant, and further on to properly plan dosage and toxicity experiments with the plant.

The data you share will not be given to other parties. Feel free to contact ukutuyai@gmail.com with any questions that may arise.

Thank you for your time!

Uyai-abasi Otuekong Ukut, sixth year medical student, University of Pécs

SECTION 1-

Collection of your basic data to help with demographic observation

- Where is your country of origin?
  - Cameroon
  - Nigeria
  - Ghana
  - Other:____
- Which region of the country are you from?
  - South-South
  - South-east
  - South-west
  - North-east
  - North-west
  - Central
  - Other:____
    ____
- Where is your area of settlement?
  - City
  - Town
  - Suburb
  - Village
  - Other
- What is your ethnic group ?

- ____
- What is your biological sex?
  - Female
  - Male
  - Other
- How old are you?
  - 0-12
  - 13-18
  - 19-25
  - 26-30
  - 31-50
  - 51-70
  - above 70
- Which is the highest level of education you have achieved?
  - Primary Education
  - Secondary school
  - Technical college
  - Bachelor Degree BD
  - PhD
  - Other
- Do you know the Bitter kola plant (*Garcinia kola Heckel)*?
  - Yes
  - No

SECTION 2: PERSONAL USAGE OF BITTER KOLA PLANT (*GARCINIA KOLA HECKEL)*

This is Information on how you consume bitter kola plant as a regular part of your lifestyle

- What part(s) of the Bitter kola plant do you eat?
  - None
  - Seeds
  - Leaves
  - Bark
  - Root
  - Other:____
- In what form do you usually use the Bitter kola part(s)

	Fresh	Dried	Boiled	Pounded	Other	None
Seeds						
Leaves						
Bark						
Root						
Other Part						

- If you answered "other" in the previous question, please give more information here!
    - ____
- If you answered "other" in the previous question, please give more information here!
    - Never
    - a few weeks
    - a few months
    - 1-3 years
    - 10 years
    - 20 years
    - more than 30 years
    - Other:____
- How long have you been taking bitter kola?
    - Never
    - a few weeks
    - a few months
    - 1-3 years
    - 10 years
    - 20 years
    - more than 30 years
    - Other:____
- Frequency of usage (How often do you consume the...

	Daily	Weekly	Every 2 weeks	Monthly	Yearly	Not used
…seeds						
…leaves						
…bark						
…other parts						

- Frequency of usage (How often do you consume the….

	Daily	Weekly	Every 2 weeks	Monthly	Yearly	Not used
…seeds						
…leaves						
...bark						
…root						
…other parts						

- If you answered "…other parts" in the previous question, please give more information here!
    - ____
- Consumption of seeds

	1-3 seeds	4-7 seeds	8-10 seeds	11-19 seeds	20-50 seeds	more than 50 seeds	none
How many do you eat in ONE SITTING ?							
What is the MAXIMUM number you eat A DAY?							

- Do you use Bitter kola seeds to welcome visitors into your home?
    - yes
    - no

SECTION 3:TRADITIONAL MEDICINAL USAGE OF BITTER KOLA PLANT

This section is to collect the medicinal knowledge you have on the bitter kola plant.

- What part(s) of the Bitter kola tree do you use to treat illness(es)?
    - Seeds
    - Leaves
    - Bark
    - Root
    - Sap
    - None
    - Other:____
- Where did you learn the medicinal use of bitter kola plant?
    - Grandparent
    - Parent
    - Sibling
    - Neighbours
    - Herbal book
    - Traditional doctor
    - Medical health officer
    - Other:____
- What conditions do you treat with bitter kola?

	Bacterial Infection	Diabetes	For General well-being	Liver problem	Respiratory issues-Cough	Stomach upset	Wound healing	Other	Not used
Seeds									
Leaves									
Bark									
Root									
Sap									
Other part									

- If you chose “other” in the previous question of you just feel it’s relevant, please give more information here!
  - ____
- In what form do you usually use or store the Bitter kola part(s) when treating illnesses?

	Fresh	Dried	Boiled	Pounded	Other	None
Seeds						
Leaves						
Bark						
Root						
Sap						
Other part						

- If you answered "other" in the previous question or you feel it’s relevant, please give more information here!
  - ____
- How much of the prepared medicine do you usually use at ONE TIME?
  - ____
- What is the MAXIMUM amount of prepared medicine do you use during one day?
  - ____

____
____
____
____

- How OFTEN do you use the treatment to cure illness(es)?

	3-5 times daily	1-2 times daily	Daily	Every 2 days	Weekly	As needed- when symptoms are seen	Not used
Bacterial Infection							
Diabetes							
For General well-being							
Liver problem							
Respiratory issues- Cough							
Stomach upset							
Wound healing							
Other							
Not used							

- How LONG do you use the treatment to cure illness(es)?

	3-5 times daily	1-2 times daily	Daily	Every 2 days	Weekly	As needed- when symptoms are seen	Not used
Bacterial Infection							
Diabetes							
For General well-being							
Liver problem							
Respiratory issues- Cough							

Stomach upset							
Wound healing							
Other							
Not used							

- Who are you treating with Bitter kola?
    - Yourself (Self-medication)
    - Your children
    - Your parent(s)
    - Community member(s)
    - Domestic animals
    - Other:____
- Did you ever experience any adverse effects of Bitter kola? If yes, please give details! (like: what was the effect? which part/form of Bitter kola, and how much caused it?)
    - ____
- If you have any other information about Bitter kola that you think might be important for my research, please tell me about it here!
    - ____
